# Artificial Neural Networks to Predict the Apparent Degree of Supersaturation in Supersaturated Lipid-Based Formulations: A Pilot Study

**DOI:** 10.3390/pharmaceutics13091398

**Published:** 2021-09-05

**Authors:** Harriet Bennett-Lenane, Joseph P. O’Shea, Jack D. Murray, Alexandra-Roxana Ilie, René Holm, Martin Kuentz, Brendan T. Griffin

**Affiliations:** 1 School of Pharmacy, University College Cork, T12 YT20 Cork, Ireland; hbennettlenane@ucc.ie (H.B.-L.); joseph.oshea@ucc.ie (J.P.O.); 117303616@umail.ucc.ie (J.D.M.); aIlie@its.jnj.com (A.-R.I.); 2 Drug Product Development, Janssen Research and Development, Johnson & Johnson, Turnhoutseweg 30, 2340 Beerse, Belgium; rholm@its.jnj.com; 3 Department of Physics, Chemistry and Pharmacy, University of Southern Denmark, DK-5230 Odense, Denmark; 4 School of Life Sciences, University of Applied Sciences and Arts Northwestern Switzerland, Hofackerstrasse 30, 4132 Muttenz, Switzerland; martin.kuentz@fhnw.ch

**Keywords:** lipid-based drug delivery, computational pharmaceutics, machine learning, supersaturated lipid-based formulations

## Abstract

In response to the increasing application of machine learning (ML) across many facets of pharmaceutical development, this pilot study investigated if ML, using artificial neural networks (ANNs), could predict the apparent degree of supersaturation (aDS) from two supersaturated LBFs (sLBFs). Accuracy was compared to partial least squares (PLS) regression models. Equilibrium solubility in Capmul MCM and Maisine CC was obtained for 21 poorly water-soluble drugs at ambient temperature and 60 °C to calculate the aDS ratio. These aDS ratios and drug descriptors were used to train the ML models. When compared, the ANNs outperformed PLS for both sLBF_Capmul_^MC^ (*r*^2^ 0.90 vs. 0.56) and sLBF_Maisine_^LC^ (*r*^2^ 0.83 vs. 0.62), displaying smaller root mean square errors (RMSEs) and residuals upon training and testing. Across all the models, the descriptors involving reactivity and electron density were most important for prediction. This pilot study showed that ML can be employed to predict the propensity for supersaturation in LBFs, but even larger datasets need to be evaluated to draw final conclusions.

## 1. Introduction

In the face of increasing pressures for accelerated development, the work of formulation scientists could be advanced through miniaturised screening tools, computational methods, and a structured approach in preclinical testing [1,2]. Currently, more conservative “tried-and-tested” approaches to formulation design are typically employed, often leading to suboptimal formulations that may disregard influential molecular and physicochemical drug properties or compound interactions with formulation excipients. However, such classical formulation development is likely to change as different computational tools are already widely used in drug discovery and are gaining momentum in pharmaceutical development. Quantity structure–activity relationships (QSARs) have streamlined the selection of candidates with optimal binding profiles [3], physiologically based pharmacokinetic (PBPK) models have aided the simulation of pharmacokinetic parameters [4], while theory- or data-driven modelling applications have improved formulation development [5,6,7,8,9,10,11,12]. Using data-driven machine learning (ML) approaches, improved success rates are achievable by ascertaining the statistical relationships between molecular descriptors and the intended response.

The main goal of predicting an outcome using input variables is the same for both partial least squares (PLS) and artificial neural network (ANN) ML algorithms. However, the mathematical approaches used differ in terms of the dimensionality reduction in data versus the potential for non-linear data fitting. PLS is a well-established multivariate regression dimensionality reduction method. The model calculates the X- and Y-matrices to find the principal components in X (independent variables) that capture most of the variance in Y (dependent variable). These initial data are projected into a latent variable space, thereby maximising the covariance between X and Y [13]. While PLS aims to find a linear (or polynomial) relationship between X and Y, ANNs represent an emerging ML algorithm. ANNs differ in their capability to detect complex non-linear X–Y relationships while detecting possible interactions between X variables [14]. ANNs mimic basic human biological information processing methods, as the structure of the multilayer perceptron (MLP) algorithm contains some main elements: input layer, hidden layer, output layer, activation functions, and connection weights. Each neuron receives signals/inputs from other neurons in the preceding layers or directly from the independent variables. This signal has an associated weighted value, which determines the strength of this interconnection. A weighted sum of these inputs is computed and transformed using an activation function to produce an output signal, which is sent to the next neurons in subsequent layers. During training, samples are passed through the network and synaptic weights are continuously adjusted until a minimum prediction error is achieved. While an in-depth analysis of ANNs can be found in the literature [15,16], current research suggests that ANNs may provide a promising alternative tool to decode complex pharmaceutical datasets.

Over the last decade, interest regarding the use of ML algorithms across diverse disciplines in pharmaceutical design and development has grown [11,17,18,19,20,21,22,23,24,25,26]. While ML models have been produced to optimise lipid-based formulation (LBF) development [3,22,27,28,29,30,31,32,33], the application of more novel ML approaches for bio-enabling formulations currently focuses on solid dispersions (SDs) [21,34,35]. However, their application to LBFs, particularly supersaturated LBFs (sLBFs), remains unexplored. LBFs, in their most utilised form of lipid solutions, aim to solubilise poorly water-soluble drugs (PWSDs) and to improve biopharmaceutical properties by simulating endogenous lipid absorption pathways [36]. However, commercial utilisation has been declining [37], likely partly attributable to the dose loading limitations given by the inherent drug solubility in the lipid vehicle [38,39]. One delivery solution has involved the development of sLBFs. These are kinetically stable solutions containing a drug concentration above the thermodynamic solubility, where increased drug loads and exposure are achieved through thermally inducing supersaturation [38,40,41]. Previously, supersaturated solutions such as sLBFs have been characterised by the apparent degree of supersaturation (aDS) ratio [42,43,44], calculated to determine the propensity of drugs to supersaturate in specific lipid systems (i.e., fold increase in drug solubility with elevation of temperature). This has been used as an indicator of the likelihood of designing sLBFs and is critical regarding the ability to maintain drug supersaturation upon storage [43]. Therefore, we hypothesise that an in silico ML model predicting aDS from molecular properties would support streamlined screening of sLBFs.

Consequently, this pilot study sought to investigate if ANN modelling could be used to predict the aDS in sLBFs using a dataset generated for 21 PWSDs. PLS regression models produced from the same dataset facilitated a comparison of the two computational techniques for this dataset. Two medium-chain (MC) and long-chain (LC)-based mono/di-glycerides formulations were chosen as mono-/di-glyceride systems that previously facilitated improved supersaturation propensity and streamlined drug-excipient screenings [38,45,46]. PLS has been previously employed in computational modelling for LBFs [29,30]. However, this study provides, to the best of our knowledge, the first investigation into the application of ANNs to predict maximum dose loading in LBFs.

## 2. Materials and Methods

### 2.1. Chemicals and Materials 

Celecoxib was purchased from Astatech Inc. (Bristol, PA, USA), while cinnarizine, JNJ-2A, ibuprofen, and itraconazole were obtained from Janssen Pharmaceutica (Beerse, Belgium). Fenofibrate and indomethacine were purchased from Sigma-Aldrich (Wicklow, Ireland). Progesterone, felodipine, sulfalazine, haloperidol, danazol, naproxen, venetoclax, carvedilol, dipyridamole, niclosamide, griseofulvin, fenofibric acid, ketoconazole, and clotrimazole were purchased from Kemprotec (Carnforth, UK), and Capmul MCM C8 was kindly donated by Abitec (Columbus, OH, USA). Maisine CC was a kind gift from Gattefossé (Lyon, France). All other chemicals and solvents were of analytical or high-performance liquid chromatography (HPLC) grade, purchased from Sigma-Aldrich (Wicklow, Ireland).

### 2.2. Formulations

Two prototype single-component LBFs were chosen based on their previous successful applications as sLBFs [43]. The MC system contained Capmul MCM, a blend of MC mono- and di-glycerides where caprylic acid (C8) is considered the predominant fatty acid. The LC system contained Maisine CC, a blend of LC mono- and di-glycerides where linoleic acid, C18:2, is considered the predominant fatty acid. These formulations are termed sLBF_Capmul_^MC^ and sLBF_Masine_^LC^ when referring to solubility testing at 60 °C.

### 2.3. Dataset Selection/Drug Physiochemical and Molecular Properties

Twenty-one structurally diverse PWSDs were selected (Table 1), where the criteria included the availability of physicochemical properties and potential utilisation as part of a commercial LBF, or a sLBF. The compounds were classified according to their glass-forming ability (GFA) [44], where eight drugs were Class 1, three drugs Class 2, and 10 drugs Class 3. Greater than 250 molecular descriptors were predicted from ADMET Predictor 9.5 (Simulations Plus, USA) and added to the experimental drug properties of melting point (T_m_), glass transition temperature (T_g_), entropy of fusion (∆*S_fus_*), enthalpy of fusion (∆*H_fus_*), T_m_/T_g_, and reduced glass transition temperature (T_rg_), obtained from the literature [9,38,47,48,49]. As the molecular properties can be obtained for any drug once the structure is known, they were used as input data.

### 2.4. Equilibrium Solubility Determination

Equilibrium drug solubility studies were conducted in both LBFs at ambient temperature (AT) (22 °C) and at an elevated temperature (60 °C). The solubility at both temperatures for cinnarizine, celecoxib, and JNJ-2A was obtained previously [43]. Solubilities for the remaining drugs were conducted using an equivalent protocol as follows: An excess amount of drug was added to 2 mL of either Capmul MCM or Maisine CC in screw cap glass vials containing a magnetic stirrer. The resulting suspensions were stirred on a stirring plate (Mixdrive 15, 2MAG, München, Germany) at 200 rpm and incubated in temperature-controlled ovens (APT.line^TM^ BD (E2), Binder, GmbH, Tuttlingen, Germany) at and 60 °C. Aliquots were sampled at 24, 48, and 72 h (or further, if required) and centrifuged at 21,380× *g* (i.e., relative centrifugal force) (Mikro 200 R, Hettich GmbH, Tuttlingen, Germany) at 22 and 40 °C, respectively, for 15 min. Daily sampling was continued until equilibrium solubility was reached, i.e., solubility between two consecutive samples differed by less than 10%. The supernatant was centrifuged under identical conditions. To solubilise the oily excipient, the supernatant was diluted 1:10 (*v/v*) in acetonitrile/ethyl acetate (1:3, *v/v*), followed by further 1:10 (*v/v*) dilution with acetonitrile/ethyl acetate (3:1, *v/v*) and a final dilution with mobile phase. The efficiency of extraction recovery was >94%, tested using a known amount of each compound. All samples were run in triplicate, and the drug concentrations were determined using an Agilent 1200 series HPLC system. The columns and HPLC testing conditions for each drug can be found in the Supplementary Materials (Table S2).

Subsequently, to assess the short-term stability upon storage at AT, following the second centrifugation step, an aliquot of supernatant from the 60 °C samples was allowed to cool at for 2 h. Then, sampling and analysis was conducted as outlined above, with values obtained presented as aDS_2h_. These short-term stability studies were conducted for the majority of the compounds.

### 2.5. Apparent Degree of Supersaturation (aDS)

The apparent degree of supersaturation (aDS), as previously defined [42], was determined as the ratio of the concentration of the drug in the supersaturated solution according to this experimental methodology and the concentration in the saturated solution. This theoretical aDS was calculated according to Equation (1) for both sLBFs loaded with drugs at 60 °C:

(1) $$aDS=C_{supersaturation}\;/S_{equilibrium,}$$

where *C_supersaturation_* is the concentration of the drug determined after heating the sLBF (to 60 °C) and *S_equilibrium_* is the equilibrium solubility at AT.

Subsequently, to facilitate comparisons of the short-term stability of the sLBFs after 2 h, a second aDS (aDS_2h_) was calculated according to Equation (2):

(2) $$aDS_{2h}=C_{supersaturation\left(2h\right)}\;/S_{equilibrium,}$$

where, in this case, *C_supersaturation(2h)_* is the drug concentration in the lipid system that was heated to 60 °C, followed by cooling to AT for 2 h. The values are reported as aDS (± standard error (SE)), with the SE calculated from Equation (3):

(3) $$\mathrm{SE}=aDS\times \sqrt{\frac{SA^{2}}{A^{2}}+\frac{SB^{2}}{B^{2}},}$$

where *A*, *B*, *SA*, and *SB* refer to the mean measured solubility values and standard errors for the equilibrium solubility at AT (A) and the concentration of the drug in the lipid system at 60 °C with/ without 2 h of cooling (B). The graphs were obtained using Prism (Version 5, Graphpad, San Diego, CA, USA).

### 2.6. Differential Scanning Calorimetry

The majority of GFA classifications and T_g_ values were obtained from the literature. However, for fenofibric acid, progesterone, and sulfasalazine, this information was obtained experimentally using differential scanning calorimetry (DSC) equipped with a TA Q1000 with a TA Refrigerated Cooling System 90 (TA Instruments, New Castle, DE, USA). The cell was purged with nitrogen at 50 mL/min. After the midpoint glass transition temperature (T_g,mid_) had been determined, crystallisation screening experiments were conducted using the protocol by Baird et al. [47]. In brief, 2 mg of drug weighed into a T-zero pan and heated at 10 °C min^−1^ to 10 °C above the T_m_ of each drug (as per Table 1, held isothermally for 3 min, cooled at a rate of 20 °C min^−1^ to −75 °C, and reheated to 10 °C at 10 °C min^–1^ above the T_m_ of each drug. Sample weights for each repeat sample were within 1 mg and experiments were run in triplicate. GFA was categorised, according to Baird et al., into Class I (in case of crystallisation during cooling prior to the T_g_), Class II (for no crystallisation during cooling, but crystallisation was observed upon reheating above T_g_), and Class III (for no crystallisation observed during cooling nor reheating to T_m_) [47].

### 2.7. Statistical Analysis

To test the significance between paired solubility values in Capmul MCM versus Maisine CC and sLBF_Capmul_^MC^ versus sLBF_Maisine_^LC^, the distribution of the differences was used to determine normality, or lack thereof. A two-sided bootstrap-paired test (5000 samples) determined the significance (*p* < 0.05). Simple scatter plots were produced for Capmul MCM versus Maisine CC and sLBF_Capmul_^MC^ versus sLBF_Maisine_^LC^, regression coefficients fitted for interpretation, and a bootstrap test for the coefficients conducted. Statistical analysis was conducted using SPSS Statistics (Version 26, IBM Corporation, Armonk, NY, USA).

### 2.8. Partial Least Squares Regression (PLS)

Quantitative prediction of aDS using PLS regression was conducted using Unscrambler (Version 11, Camo Analytics, Bedford, MA, USA). PLS model development followed the standard steps described previously [30]. Molecular structures were acquired as smiles from PubChem and used as inputs for the ADMET Predictor (Version 9.5, Simulations Plus, Lancaster, CA, USA) to calculate >250 molecular descriptors, which were added to T_m_, T_g_, ∆H*_fus_*, ∆S*_fus_*, T_m_/T_g_, and T_rg_ and used as variable inputs. The individual modelling responses were aDS ratios from both sLBF_Capmul_^MC^ and sLBF_Maisine_^LC^. Principal component analysis (PCA) was applied for a randomised assignment of training/test data. The training set criteria were that it covered the chemical space of the test set, along with a relatively even spread of aDS ratios. A Hotelling’s T^2^ ellipse was applied for outlier detection (95% confidence interval). The nonlinear iterative partial least squares (NIPALs) algorithm was utilised, and all variables were mean-centred, de-identified, and standardised through scaling by standard deviation. To limit the overfitting potential, a limit of two principal components was used. Variable reduction was performed as previously described [30] using Martens’ uncertainty test [50], an important variables plot, and correlation loadings plot. Model accuracy was validated by the root mean square error (RMSE) of the training and test sets.

### 2.9. Artificial Neural Networks (ANNs)

Multilayer perception artificial neural networks (MLP-ANNs) were produced using SPSS Statistics (Version 26, IBM Corporation, Armonk, NY, USA) to predict aDS. A partition variable using the same training/test set split was utilised to compare PLS versus ANNs. The input properties were obtained as described above and were rescaled through standardisation, where values were converted to their z-scores. A hyperbolic tangent was chosen as the activation function for the hidden layer, while an identity output function was used in the output layer [51]. Supervised learning using the scaled conjugate gradient (SCG) algorithm was chosen for its speed, and a lack of user-critical parameters [52]. Batch training was selected due to the relatively small dataset size and the learning algorithm employed. Variable reduction was initially conducted using an independent variable importance analysis. As an arbitrary criterion, only variables with a relative importance of >70% were included in the architecture going forward. Topologies with only one hidden layer were considered to avoid overfitting. The optimum number of neurons in the hidden layer was identified following a systematic trial-and-error approach, where the number of neurons in the hidden layer were manually altered between 2 and 20, with runs performed in triplicate. The optimal network size was chosen thorough minimum RMSE in the training and test sets. The most important variables in each network were elucidated from the normalised importance chart. The PLS and ANN models produced were directly compared in terms of different performance evaluation functions, including correlation coefficient (r^2^), training set RMSE, test set RMSE, and residuals by predicted charts.

## 3. Results

### 3.1. Comparing the Solubility of MC- and LC-based LBFs and sLBFs

**Figure 1:**
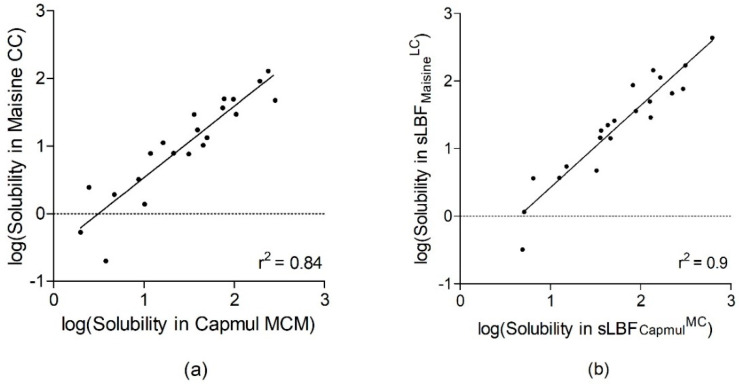
Scatter plots of the solubility in Capmul MCM versus Maisine CC (**a**) and sLBF_Capmul_^MC^ versus sLBF_Maisine_^LC^ (**b**). Formulation abbreviations can be inferred from the main text.

**Figure 2:**
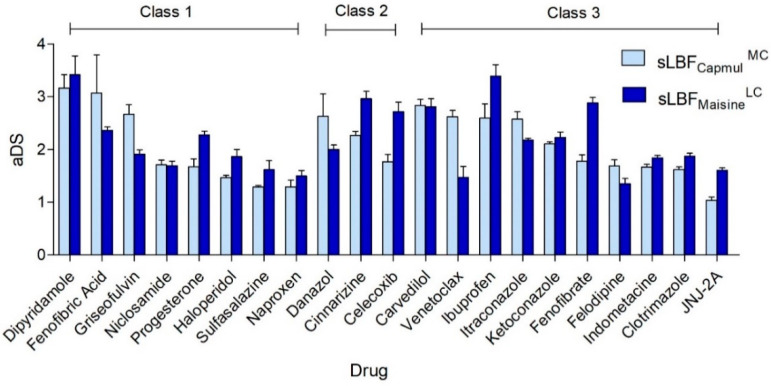
Apparent degree of supersaturation (aDS) ratios achieved for the dataset in both sLBF_Capmul_^MC^ and sLBF_Maisine_^LC^. No clear aDS trend was elucidated in terms of the glass-forming ability (GFA) classification (as grouped). Details and definitions of the abbreviations are given in the text.

The initially solubility in both LBFs (Capmul MCM and Maisine CC) at AT and both sLBFs (sLBF_Capmul_^MC^ and sLBF_Maisine_^LC^) at 60 °C was compared. Significant differences were seen at AT (* *p* < 0.05) and at 60 °C (* *p* < 0.05). The beta coefficients of the regression lines of both Maisine CC versus Capmul MCM and sLBF_Maisine_^LC^ versus sLBF_Capmul_^MC^ were also significant (both * *p* < 0.05). A relatively strong correlation was established between solubility (logS) in both blends at AT (*r*^2^ = 0.84). This was stronger at 60 °C (*r*^2^ = 0.90) (Figure 1). Fourteen of the 21 (66%) drugs demonstrated a higher aDS ratio in sLBF_Maisine_^LC^ versus sLBF_Capmul_^MC^ (Figure 2). All 21 drugs showed higher solubility in Capmul MCM when compared to Maisine CC at AT. In general, this trend was repeated at 60 °C, except for fenofibrate and cinnarizine, where the order of solubility was switched, albeit not significantly so.

### 3.2. Apparent Degree of Supersaturation

Increases in thermally induced solubility were seen for all drugs in both the MC and LC sLBFs (aDS ratio >1), reflecting increased dose loading relative to conventional LBFs. Drug solubility in Capmul MCM, Maisine CC, sLBF_Capmul_^MC^, and sLBF_Maisine_^LC^ are presented as mean ± SD (*n* = 3) in the Supplementary Materials (Table S1). The extent of aDS ranged from 1.04 to 3.17 in sLBF_Campul_^MC^ and between 1.06 and 3.4 in sLBF_Maisine_^LC^ (Figure 2). In the rank order of supersaturation propensity, the investigational drug candidates JNJ-2A and felodipine produced the lowest aDS in sLBF_Capmul_^MC^ and sLBF_Maisine_^LC^, respectively. Dipyridamole demonstrated the highest aDS using both sLBFs. 

While correlations between GFA class and aDS ratios have previously been observed using solvent shift-mediated supersaturation [42], our data revealed no clear trend between aDS and GFA (Figure 2). The mean aDS for sLBF_Capmul_^MC^ and sLBF_Maisine_^LC^ in each GFA class was 2.04 and 2.08 (class 1), 2.22 and 2.56 (class 2), and 2.05 and 2.16 (class 3), respectively, indicating that between GFA classes, no significant differences were seen. The mean aDS for the three GFA classes also did not significantly differ according to the sLBFs’ fatty acid chain length. 

Upon comparison of the aDS values obtained after cooling of the 60 °C samples for 2 h at AT (aDS_2h_), average differences in aDS ratio units of 0.17 (sLBF_Capmul_^MC^) and 0.16 (sLBF_Maisine_^LC^) were observed (Supplementary Materials Table S3), corresponding to average drug solubility losses of 7.9% and 7.7% upon cooling, respectively. For this dataset, which comprised drugs of a variety of chemical structures, the range of precipitation upon removal of heating was moderate, i.e., less than 20%, with 71% of drugs displaying a less than 10% loss after 2 h.

### 3.3. Quantitatively Predicting aDS Using PLS and ANNs

**Figure 3:**
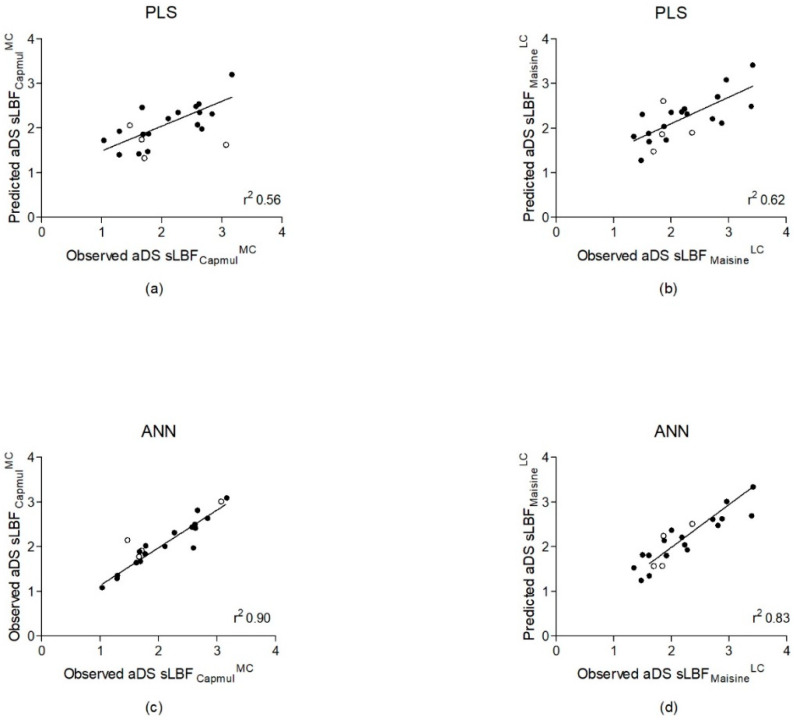
Scatter plots illustrating the predicted versus observed aDS values obtained for aDS sLBF_Capmul_^MC^ using PLS (*r*^2^ = 0.56) and ANNs (*r*^2^ = 0.90) (**a**,**c**). Scatter plots illustrating the predicted versus observed aDS values obtained for aDS sLBF_Maisine_^LC^ using PLS (*r*^2^ = 0.62) and ANNs (*r*^2^ = 0.83) (**b**,**d**).

Quantitative models predicting aDS were produced using PLS and ANNs. Unabridged versions of all the drug descriptor abbreviations in this section can be found in the Supplementary Materials (Figure S1). PLS models for both aDS sLBF_Capmul_^MC^ and aDS sLBF_Maisine_^LC^ of two PCs and eight and nine input variables, respectively, were developed (Table 2). The aDS sLBF_Capmul_^MC^ model produced relatively weak predictions of *r*^2^ = 0.56, and in the training and test sets, the RMSE was 0.4 and 0.79 using eight variables: VMcGowans, N_Hydrgn, EEM_Afc, EEM_AFnp, SHCH_321, SHaaCH, EEM_NFc, and Pi_FMi4 (Figure 3). Martens’ uncertainty test designated SHCH_321 and EEM_NFc as the most important variables. Comparatively, the two PCs aDS sLBF_Maisine_^LC^ PLS model displayed a correlation coefficient of *r*^2^ = 0.62 and an RMSE in the training and test sets of 0.4 and 0.45, respectively, using nine input variables: HIVI-TC, N_FrRotB, NPA_Q2, EEM_Nfc, EEM_NFnp, Pi_Aqo, Pi_AQc, Pi_FPI3, and Pi_FMi6. In this case, N_FrRotB and Pi_FMi6 were the most important variables. 

**Figure 4:**
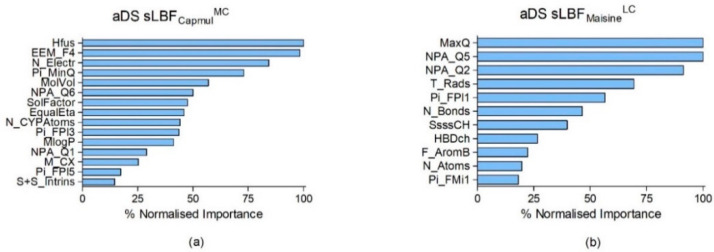
Normalised importance charts of the ANNs for sLBF_Capmul_^MC^ (**a**) and sLBF_Maisine_^LC^ (**b**) detailing the percentage importance of the input variables in predicting aDS. Details and explained abbreviations are given in the main text and Supplementary Materials (Figure S1).

Using ANNs, MLP 15-5-1 for sLBF_Capmul_^MC^ and MLP 11-8-1 for sLBF_Maisine_^LC^ were produced (Table 2). These equated to input layers with 15 and 11 drug properties, one hidden layer of five and eight nodes, and singular output layers, i.e., predicted aDS. A strong correlation between the predicted and observed aDS values was observed for the sLBF_Capmul_^MC^ network (*r*^2^ = 0.90) (Figure 3). This demonstrated a low RMSE upon training (0.19) and testing (0.36) (Table 2). The properties included in the network were Pi_FPl5, SolFactor, N_CYPAtoms EEM_F4, Pi_FPl3, NPA_Q6, MlogP, MolVol, NPA_Q1, S+S_Intrins, EqualEta, ∆Hfus, M_CX, Pi_MinQ, and N_Electr. The normalised importance chart signified ∆H*_fus_*, EEM_F4, and N_Electr as the three most significant variables (Figure 4). The predicted and observed aDS values for aDS sLBF_Maisine_^LC^ were strongly correlated (*r*^2^ = 0.83), as training and testing RMSEs of 0.28 and 0.25 were observed (Figure 3). The drug properties in the final network were N_Bonds, Pi_FPl1, T_Rads, MaxQ, N_Atoms, Pi_FMi1, HBDch, F_AromB, NPA_Q2, SsssCH, and NPA_Q5. MaxQ, NPA_Q5, and NPA_Q2 were the most important variables (Figure 4).

Upon model comparison, the ANNs produced improved aDS predictions for both sLBFs, as both ANN models displayed substantially stronger correlation coefficients, lower training and testing RMSEs, and smaller residuals. The residuals for both ANN models demonstrated almost complete independence and random distribution in residuals by predicted charts (Supplementary Materials Figure S2). The relatively poor performance of the PLS models indicates that their inclusion was primarily for the purpose of comparison with the ANNs.

## 4. Discussion

The increasing adoption of model-based approaches across drug design and development has aided in improving efficiency in pharmaceutical research. Computational tools exist across the pharmaceutical industry in many forms. However, for LBFs, thus far, the drug property-based aspects of computational pharmaceutics have focused on solubility predictions for traditional solution or self-emulsifying drug delivery system (SEDDS) formulations [27,28,29,30]. The exploration of ANNs to support LBF development remains relatively unexplored. As a result, the main purpose of this research was to investigate if an ANN model could be developed to predict the aDS in sLBFs using drug physicochemical or molecular properties. These predictions could be used to guide whether the degree of supersaturation in lipids is sufficient to enable dosing in early development.

Accordingly, as part of this pilot study, two ANNs were developed, which predicted aDS in sLBFs from their drug properties. These ANNs produced superior predictions compared to PLS models developed using the same available dataset. These ANNs predicting aDS (sLBF_Capmul_^MC^ and sLBF_Maisine_^LC^), containing one hidden layer of five and eight nodes and using fifteen and eleven drug properties, respectively, yielded strong prediction accuracy performance (*r*^2^ = 0.90, 0.83) and low RMSEs upon both training (0.19, 0.28) and testing (0.36, 0.25). In comparison, when using PLS, a lower accuracy of prediction (*r*^2^ = 0.56, 0.62) and higher residuals and RMSEs upon training (0.4, 0.4) and testing (0.79, 0.45) were observed using eight and nine drug properties. Accordingly, this study demonstrates that ANNs can be applied to link molecular drug properties to a predicted maximum dose loading capacity, i.e., aDS upon thermal induced supersaturation.

These modelling results suggest that aDS prediction is a complex and multifaceted phenomenon, as for this dataset numerous drug descriptors and non-linear mathematical algorithms were required for higher accuracy. One explanation for the improved performance of ANNs for this dataset may be attributed to its capability in decoding multidimensional highly non-linear relationships in datasets in the hidden layer, as opposed to linear relationships of the latent variables obtained through PLS. Consequentially, this work highlights the capability of ANNs to provide an industrially applicable alternative to the more established computational pharmaceutics modelling methods such as PLS. While PLS regression has advantages versus ANNs in terms of model transparency and decreased complexity in interpretation, in situations of interrelationships or substantial non-linearity, as seen here, ANNs may improve the accuracy of prediction. Therefore, it is hoped that this pilot study can initiate future larger-scale studies to strengthen these predictions. 

Modelling indicated that drug properties hold key information about aDS. Overall, a wide range of drug descriptors, reflecting topology, reactivity, structure and size, electrostatics, and thermodynamics, were significant. Trends in important properties were revealed. The three most important properties predicting aDS for sLBF_Capmul_^MC^ were ∆H*_fus_* (enthalpy of fusion), EEM_F4 (fourth component of the autocorrelation vector of sigma Fukui indices), and N_Electr (total number of electrons in a molecule) (Figure 4). ∆H*_fus_* is a thermodynamic property, involving the amount of thermal energy which must be absorbed or evolved to change 1 mole of a solid to a liquid with no temperature change [53]. ∆H*_fus_* was shown to previously inversely correlate with the potential of a drug to supersaturate from solvent shift-induced supersaturation [42]. Fukui indices are frontier orbital indices, indicating atomic electron affinity and a molecule’s ability to become polarised upon changes to electron density [54,55]. Similar Fukui indices were previously important properties governing the intrinsic dissolution rate of PWSDs in biorelevant media [56] and in support vector machine modelling to predict GFA for compounds between 200 and 300 g/mol. In this case, a high value, which denoted increased electron reactivity, suggested a non-glass former [9]. The number of electrons in a molecule is related to its reactivity, as the electrons in the outermost atom shell determine the reactivity. Generally, polarizability increases as the volume occupied by electrons increases. To predict aDS in sLBF_Maisine_^LC^, MaxQ (maximal PEOE partial atomic charge), NPA_Q5, and NPA_Q2 (fifth and second components of the autocorrelation vector of estimated NPA partial atomic charges) were the most significant properties (Figure 4). Both natural population analysis (NPA) and partial equalization of orbital electronegativity (PEOE) are methods to calculate partial atomic charges. They describe the charge and electron density distributions within molecules, providing clues about chemical behaviour [57,58]. Comparatively, the PLS performance was poor in terms of correlation and residual error, and therefore, PLS is more suited here as a qualitative model. The fact that PLS and ANNs use different mathematical approaches to obtain correlations, and that ANNs can incorporate interrelationships between descriptor variables, likely explains the differences in the final model variables. Despite the observed differences, the Fukui indices, partial atomic charges, and atom-type E-state indices were significant for PLS and ANN prediction, supporting their importance for aDS.

As a lack of thermodynamic stability is a fundamental limitation of sLBFs, it is imperative that supersaturation is maintained over a sufficient period to facilitate adequate absorption. In this study, after 2 h of cooling, the sLBFs maintained relatively high levels of supersaturation across a variety of drugs, i.e., >80% of the drug remained above saturation solubility. aDS was previously suggested as a guide for the likelihood of precipitation from sLBFs [43], where drugs that generated higher aDS coupled with high T_m_/T_g_ ratios (higher crystallisation tendency) demonstrated quick precipitation on storage at 25 °C, while drugs with low aDS and low T_m_/T_g_ ratios resulted in good storage stability. Similarly, in this study, Dipyridamole (a Class 1 GFA drug with a high T_m_/T_g_ ratio and ∆S*_fus_*) produced the highest aDS in both sLBFs, while Class 3 GFA JNJ-2A and felodipine, both possessing low crystallisation tendencies produced the lowest aDS. Therefore, this could provide an extended application of these models to anticipate the precipitation potential, with reference to the indicators of crystallisation tendency (T_m_/T_g_, ∆S*_fus_*) [47]. However, investigations regarding the overall accuracy of this combination were not within the scope of this current pilot study. 

The influence of fatty acid chain length in terms of both aDS and drug solubility between the MC- and LC-based mono/di-glyceride blends was also observed. Similarly, to previous work involving MC and LC triglycerides [29,30], a relatively strong correlation was found between solubility in both blends at AT. Interestingly, it appeared that the common effect of heating became more influential for solubility rather than the properties of the lipids, as heating increased the strength of the correlation. While the solubility was higher in sLBF_Capmul_^MC^ for the majority of drugs, approximately 60% demonstrated higher aDS in sLBF_Maisine_^LC^. This was potentially aided by the generally lower drug solvation in the long-chain formulation at AT, thereby permitting higher aDS gains upon heating.

Finally, as recent expert commentary has emphasised various shortcomings of data-driven modelling [11], we acknowledge the dataset used in model development here is limited in size (Supplementary Materials—Modelling Database). As such, this work was essentially a pilot study seeking to investigate the potential of ANNs to improve the accuracy of predictive models. Accordingly, the authors support strategies for further research using a larger dataset to confirm the correlations obtained and have provided the ANN models as predictive model markup language (PMML) in the Supplementary Materials (LC PMML and MC PMML). This will further clarify which molecular properties are significant for aDS, extending the applicability of the models. Notwithstanding this limitation, this pilot study successfully achieved the intended goal of demonstrating the robust predictive power of ANNs to LBF datasets. 

## 5. Conclusions

This pilot study explored the application of ANNs as a computational technique to predict aDS in sLBFs. The ANN models demonstrated accuracy in the quantitative prediction of the aDS ratios versus PLS models from the same dataset. These models, while demonstrating ANNs’ ability to capture complex data relationships, also facilitated greater insight into the relationship between drug properties and supersaturation propensity. It was revealed that this complex phenomenon is related to the molecular descriptors of electron density and chemical reactivity. The study impacts support the application of ML-based computational pharmaceutics in early LBF development testing. Future research with larger datasets will be needed to confirm this pilot study’s findings. Moving forward, integration and dissemination of computational expertise and in silico tools will be vital for efficient decision-making in the development of lipid-based drug delivery systems of the future.

## Figures and Tables

**Table 1:** Selection of the physicochemical and molecular properties of the investigated compounds collated from the literature, predicted from ADMET Predictor 9.5 or obtained experimentally using DSC. AMPH refers to ampholyte.

Drug Compound	MW (g/mol)	clogP	logD_6.5_	Acid/ Base/ Neutral	GFA Class	T_m_ (°C)	T_g_ (°C)	∆H*_fus_* (kJ/mol)	∆S*_fus_* × 0.01 (kJ/mol/K)	T_m_/T_g_	T_rg_	HBA	HBD	Rotatable Bonds
Carvedilol	406.49	3.88	2.36	B	III	114.5	41.9	53.00	13.67	1.23	0.81	5	3	10
Celecoxib	381.38	3.81	3.81	A	II	163	58	34.10	7.80	1.32	0.76	4	1	2
Cinnarizine	368.53	4.92	3.98	B	II	121	8.5	37.50	9.50	1.39	0.72	2	0	5
Clotrimazole	344.85	5.08	5.06	B	III	148	30	33.34	7.97	1.39	0.72	1	0	4
Danazol	337.47	4.26	4.26	N	II	225.5	88.3	35.50	7.12	1.38	0.73	3	1	1
Dipyridamole	504.64	3.11	3.02	B	I	163	40.4	72.00	16.51	1.39	0.72	12	4	12
Felodipine	384.26	5.03	5.03	B	III	145	45	30.98	7.38	1.31	0.76	5	1	4
Fenofibrate	360.84	5.20	5.20	N	III	79	−19	33.00	9.32	1.39	0.72	4	0	5
Fenofibric acid	318.76	3.98	1.25	A	I	184	35.4	99.00	21.66	1.48	0.68	4	1	3
Griseofulvin	352.77	2.51	2.51	N	I	245	89	39.12	7.96	1.36	0.73	6	0	3
Haloperidol	375.87	3.82	2.06	B	I	148	33	54.26	12.80	1.38	0.73	3	1	5
Ibuprofen	206.29	3.64	1.69	A	III	77	−45	26.50	7.56	1.54	0.65	2	1	4
Indometdacin	357.80	4.03	1.45	A	III	161	45	37.60	8.64	1.37	0.73	4	1	3
Itraconazole	705.65	4.89	4.89	B	III	168	58	57.60	13.00	1.33	0.75	9	0	10
JNJ-2A	498.90	5.40	5.40	N	III	142	91.2	22.90	5.50	1.14	0.88	4	3	7
Ketoconazole	531.44	3.67	3.51	B	III	146	45	52.85	12.50	1.32	0.76	7	0	8
Naproxen	230.27	3.21	1.10	A	I	152	5.9	25.65	6.03	1.52	0.66	3	1	3
Niclosamide	327.13	4.03	4.02	A	I	230	86	40.70	8.01	1.40	0.71	5	2	2
Progesterone	314.47	3.94	3.94	N	I	130	55.2	23.67	5.87	1.23	0.81	2	0	1
Sulfalazine	398.40	3.15	−0.35	A	I	245	54.6	99.00	20.08	1.58	0.63	9	3	3
Venetoclax	868.46	6.68	6.54	AMPH	III	138	64	18.40	4.50	1.22	0.82	12	3	11

**Table 2:** Overview of the ANNs produced to predict aDS for sLBF_Capmul_^MC^ and sLBF_Maisine_^LC^ from their drug properties, including their architecture and various performance indicators. Tr and Te refer to the training and test sets.

Y Variable	Model Type	Architecture	Input Variables	*r* ^2^	RMSE Tr	RMSE Te
aDS sLBF_Capmul_^MC^	PLS	2 PCs	VMcGowan, N_Hydrogn, SHCH_321, SHaaCH, EEM_Afc, EEM_Afnp, EEM_NFc, and Pi_FMi4	0.56	0.40	0.79
aDS sLBF_Capmul_^MC^	ANN	1 hidden layer, 5 nodes	Pi_FPl5, NPA_Q6, ∆H_fus_, EEM_F4, EqualEta, M_CX, MlogP, MolVol, N_CYPAtoms, N_Electr, NPA_Q1, Pi_FPl3, Pi_MinQ, S+S_Intrins, and SolFactor	0.90	0.19	0.36
aDS sLBF_Maisine_^LC^	PLS	2 PCs	HIVI-TC, N_FrRotB, NPA_Q2, EEM_Nfc, EEM_NFnp, Pi_AQo, Pi_AQc, Pi_FPI3, and Pi_FMi6	0.62	0.40	0.45
aDS sLBF_Maisine_^LC^	ANN	1 hidden layer, 8 nodes	F_AromB, HBDch, MaxQ, N_Atoms, N_Bonds, NPA_Q2, NPA_Q5, Pi_FMi1, Pi_FPl1, SsssCH, and T_Rads	0.83	0.28	0.25

## Data Availability

The database used for model development in this study is available in the Supplementary Materials (Modelling Database).

## Supplementary Materials

The following are available online at https://www.mdpi.com/article/10.3390/pharmaceutics13091398/s1, Table S1: Equilibrium solubility values and aDS for the dataset of 21 drugs using Capmul MCM and Maisine CC; Table S2: RP-HPLC/UV methods utilised in this study; Table S3: Equilibrium solubility and aDS_2h_ in sLBF_Capmu_l^MC^ and sLBF_Maisine_^LC^ and the aDS ratio difference from the average aDS and the average aDS_2h_ used to investigate the short-term stability of the sLBF after cooling at AT; Figure S1 Unabridged abbreviations of the independent input variables used in the final PLS and ANN models; Figure S2: Predicted by residual plots for sLBF_Capmul_^MC^ and sLBF_Maisine_^LC^ using PLS and ANN modelling. Modelling Database: Database containing the drug molecular descriptors used in model development. LC PMML and MC PMML: XML-format files containing the two ANN models produced in the study to predict aDS in PMML.
