# Supplementary material for: Artificial Neural Networks to Predict the Apparent Degree of Supersaturation in Supersaturated Lipid-Based Formulations: A Pilot Study
Source: Pharmaceutics. 2021 Sep 5;13(9):1398. doi: 10.3390/pharmaceutics13091398 (PMC8466847; doi:10.3390/pharmaceutics13091398)
Supplement: Supplementary file 1 [file pharmaceutics-13-01398-s001.zip › pharmaceutics-1360502-supplementary-layout (1).pdf]

pharmaceutics

Supplementary Materials: Artificial Neural Networks to Predict the Apparent Degree of Supersaturation in Supersaturated Lipid-Based Formulations: A Pilot Study.

Harriet Bennett-Lenane, Joseph P. O'Shea, Jack D. Murray, Alexandra-Roxana Ilie, René Holm, Martin Kuentz and Brendan T.

Table S1. Equilibrium solubility values (μg/mL) and aDS for the dataset of 21 drugs using Capmul MCM and Maisine CC. AT refers to Ambient Temperature. Data in brackets refer to standard deviation (SD) of solubility value or standard error (SE) of aDS ratios. * denotes data obtained from a previous publication (1).

Drug Compound	AT Solubility Capmul MCM	AT Solubility Maisine CC	Solubility 60ºC $sLBF_{Campul}^{MC}$	Solubility 60ºC $sLBF_{Maisine}^{LC}$	aDS $sLBF_{Capmul}^{MC}$	aDS $sLBF_{Maisine}^{LC}$
Carvedilol	45.07 (2.94)	10.28 (0.70)	127.90 (2.77)	28.91 (1.79)	2.84 (0.11)	2.81 (0.15)
Celecoxib	49.78 (5.61)*	13.30 (1.11)*	88.10 (6.67)	36.16 (2.83)	1.77 (0.14)	2.72 (0.18)
Cinnarizine	35.97 (1.03)*	29.27 (1.01)*	81.67 (3.98)	86.64 (6.54)	2.27 (0.07)	2.96 (0.14)
Clotrimazole	192.06 (5.91)	91.05 (3.73)	311.16 (13.89)	170.82 (5.27)	1.62 (0.05)	1.88 (0.06)
Danazol	16.32 (4.52)	11.19 (0.64)	42.91 (1.85)	22.41(0.95)	2.63 (0.43)	2.00 (0.08)
Dipyridamole	10.17 (0.98)	1.38 (0.17)	32.22 (3.12)	4.71 (0.61)	3.17 (0.25)	3.42 (0.36)
Felodipine	74.41 (4.63)	36.79 (1.04)	125.73 (12.40)	49.71 (6.19)	1.69 (0.11)	1.35 (0.10)
Fenofibrate	76.95 (6.66)	50.11 (1.38)	136.98 (11.10)	144.38 (8.82)	1.78 (0.12)	2.88 (0.11)
Fenofibric acid	11.81 (4.77)	7.80 (0.26)	36.28 (1.55)	18.45 (0.56)	3.07 (0.72)	2.37 (0.06)
Griseofulvin	4.70 (0.51)	1.92 (0.01)	12.55 (0.52)	3.68 (0.26)	2.67 (0.18)	1.91 (0.08)
Haloperidol	31.37 (1.34)	7.63 (0.94)	46.03 (1.43)	14.22 (0.45)	1.47 (0.04)	1.86 (0.14)
Ibuprofen	237.34 (14.85)	128.67 (4.05)	616.10 (103.62)	436.41 (46.36)	2.60 (0.27)	3.39 (0.22)
Indometacine	21.31 (0.70)	7.84 (0.30)	35.53 (1.55)	14.45 (0.25)	1.67 (0.05)	1.84 (0.04)
Itraconazole	1.99 (0.02)	0.53 (0.01)	5.12 (0.49)	1.16 (0.03)	2.57 (0.14)	2.18 (0.03)
JNJ-2a	283.07 (15.58)*	47.50 (1.24)	293.35 (27.73)	76.48 (2.92)	1.04 (0.07)	1.61 (0.04)
Ketoconazole	104.92 (3.32)	29.62 (0.25)	221.22 (2.98)	66.14 (5.06)	2.11 (0.04)	2.23 (0.10)
Naproxen	39.21 (4.83)	17.27 (1.98)	50.73 (6.04)	25.90 (0.45)	1.29 (0.13)	1.50 (0.10)
Niclosamide	8.73 (0.55)	3.21 (0.25)	14.96 (0.96)	5.44 (0.22)	1.71 (0.09)	1.70 (0.09)
Progesterone	97.92 (9.08)	49.49 (0.84)	163.96 (19.86)	112.75 (6.06)	1.67 (0.15)	2.28 (0.07)
Sulfalazine	3.79 (0.08)	0.20 (0.03)	4.89 (0.13)	0.32 (0.03)	1.29 (0.03)	1.62 (0.17)
Venetoclax	2.45 (0.41)	2.44 (0.16)	6.43 (0.28)	3.62 (0.84)	2.62 (0.12)	1.48 (0.21)

Table S2. RP-HPLC/UV Methods utilised in this study. ACN refers to Acetonitrile, MeOH refers to Methanol, TFA refers to Trifluoroacetic Acid, H_2O refers to Water and NaAC refers to Sodium Acetate. * denotes the fact that the mobile phase was adjusted to pH 2.5 with 5 %v/v Orthophosphoric acid.

Drug	Column	A	B	Ratio	Temp (°C)	Flow Rate (ml/min)	Inj. Vol (µL)	λ (nm)
Danazol	Symmetry C18 5 µm, 4,6 x 150 mm	ACN	H_2O	55:45	25	1	50	286
Ketoconazole	Symmetry C18 5 µm, 4,6 x 150 mm	Phosphate buffer 10 mM, pH 8.5	ACN	40:60	25	0.8	50	297
Venetoclax	Zorbax Eclipse Plus-C18 column (5 µm, 4.6 mm x 150 mm) including Zorbax 156 Eclipse Plus-C18 guard column (5 µm, 4.6 mm x 12.5 mm)	ACN + 0.5 % TFA	H20 + 0.5 % TFA	53:47	40	1	50	316
Carvedilol	Symmetry C18 5 µm, 4,6 x 150 mm	ACN	NaAc 25 mM, pH 5.0	60:40	25	1	50	280
Clotrimazole	Symmetry C18 5 µm, 4,6 x 150 mm	MeOH	ACN	95:5	25	1	50	255
Griseofulvin	Symmetry C18 5 µm, 4,6 x 150 mm	ACN	H_2O	55:45	25	1	50	292
Dipyridamole	Gemini 5µ C18 4,6 x 250 mm	ACN	H_2O	60:40	40	1	20	282
Felodipine	Gemini 5µ C18 4,6 x 250 mm	ACN	H_2O	70:30	25	1	20	360
Haloperidol	Symmetry C18 5 µm, 4,6 x 150 mm	ACN:MeOH	NaAc 25 mM, pH 5.0	65:35	25	0.8	50	247
Naproxen	Symmetry C18 5 µm, 4,6 x 150 mm	ACN + 0.1% TFA	H_2O + 0.1% TFA	60:40	25	1	50	254
Niclosamide	Symmetry C18 5 µm, 4,6 x 150 mm	95:5:0.1 ACN:H_2O:TFA	5:95:0.1 ACN:H_2O:TFA	75:25	25	1	50	332
Progesterone	Symmetry C18 5 µm, 4,6 x 150 mm	ACN	NaAc 25 mM, pH 5.5	85:15	25	1	50	254
Sulfalazine	Symmetry C18 5 µm, 4,6 x 150 mm	ACN:H_2O:TFA	H_2O:ACN:TFA	60:40	25	1	50	357
Fenofibric Acid	Symmetry C18 5 µm, 4,6 x 150 mm	ACN*	H20*	70:30	25	1	20	286

Table S3. Equilibrium solubility and aDS_{2h} in $sLBF_{Capmul}^{MC}$ and $sLBF_{Maisine}^{LC}$ and the aDS ratio difference from average aDS and average aDS_{2h} used to investigate the short term stability of the sLBF after cooling at AT.

	**Solubility 60ºC (+2h) Capmul MCM**	**Solubility 60ºC (+2h) Maisine CC**	**aDS_{2h} $sLBF_{Capmul}^{MC}$**	**aDS_{2h} $sLBF_{Maisine}^{LC}$**	**aDS Ratio Unit Change**	**aDS Ratio Unit Change**
Carvedilol	116.44 (6.57)	27.75 (1.02)	2.58 (0.13)	2.70 (0.12)	0.25	0.11
Celecoxib	101.8 (0.0)	39.6 (0.0)	2.04 (0.13)	2.97 (0.14)	0.27	0.25
Cinnarizine	100.1 (0.0)	97.5 (0.0)	2.78 (0.05)	3.33 (0.07)	0.51	0.34
Clotrimazole	308.30 (1.28)	172.73 (0.90)	1.61 (0.03)	1.74 (0.04)	0.01	0.13
Danazol	39.99 (3.54)	22.57 (1.88)	2.45 (0.41)	2.02 (0.12)	0.18	0.01
Dipyridamole	29.55 (4.25)	4.53 (0.49)	2.91 (0.29)	3.29 (0.31)	0.26	0.13
Felodipine	125.19 (5.94)	45.89 (9.05)	1.68 (0.08)	1.25 (0.14)	0.01	0.10
Fenofibric Acid	32.74 (3.17)	17.86 (0.93)	2.77 (0.66)	2.29 (0.08)	0.30	0.08
Griseofulvin	12.21 (0.15)	3.70 (0.24)	2.59 (0.16)	1.92 (0.07)	0.07	0.01
Haloperidol	45.37 (0.24)	13.47 (0.79)	1.45 (0.04)	1.76 (0.14)	0.02	0.10
JNJ-2A	259.5 (0.0)	65.8 (0.0)	0.92 (0.03)	1.38 (0.02)	0.12	0.23
Ketoconazole	224.87 (11.29)	63.82 (3.81)	2.14 (0.07)	2.15 (0.08)	0.03	0.08
Naproxen	45.42 (7.01)	20.39 (2.82)	1.16 (0.13)	1.18 (0.12)	0.14	0.32
Niclosamide	14.06 (1.02)	5.13 (0.23)	1.61 (0.09)	1.60 (0.08)	0.10	0.10
Progesterone	133.30 (16.12)	99.09 (7.27)	1.36 (0.12)	2.00 (0.09)	0.31	0.28
Sulfalazine	4.69 (0.23)	0.30 (0.03)	1.24 (0.04)	1.49 (0.16)	0.05	0.13
Venetoclax	7.13 (1.29)	2.89 (0.52)	2.91 (0.32)	1.18 (0.13)	0.29	0.30

Figure S1. Unabridged Abbreviations of Independent Input Variables used in the final PLS and ANN models. Extra information about the descriptors used can be found at this reference (9).

Variables in PLS model predicting aDS $sLBF_{Capmul}^{MC}$:

- **VMcGowan:** Mc Gowan's characteristic volume (2) .
- **N_Hydrogn:** Number of hydrogens**.**
- **SHCH_321:** Atom-type hydrogen E-state index for -CH3, -CH2- and >CH- groups (saturated aliphatic carbon) (3).
- **EEM_Afc:** Sum of absolute values of scaled sigma Fukui indices on C (4, 5).
- **EEM_AFnp:** Sum of absolute values of scaled sigma Fukui indices on nonpolar atoms.
- **EEM_NFc:** Minimum scaled sigma Fukui index on C.
- **SHaaCH:** Atom-type hydrogen E-state index for aCHa groups (aromatic carbons).
- **Pi_FMi4:** Fourth component of the autocorrelation vector of scaled pi Fukui- indices (electrophilic).

Variables in PLS model predicting aDS $sLBF_{Maisine}^{LC}$:

- **EEM_NFc**: Minimum scaled sigma Fukui index on C.
- **EEM_NFnp**: Minimum scaled sigma Fukui index on nonpolar atoms.
- **HIVI-TC:** pIC50 in log(mol/L) for inhibition of the HIV-1 Integrase 3'-processing.
- **N_FrRotB**: Number of freely rotatable bonds.
- **NPA_Q2**: Second component of the autocorrelation vector of NPA partial atomic charges.
- **Pi_AQo**: Sum of absolute values of pi partial atomic charges on O.
- **Pi_AQc:** Sum of absolute values of pi partial atomic charges on C.
- **Pi_FPI3:** Third component of the autocorrelation vector of scaled pi Fukui+ indices (nucleophilic)
- **Pi_FMi6:** Sixth component of the autocorrelation vector of scaled pi Fukui- indices (electrophilic).

Variables in MLP-ANN Predicting aDS $sLBF_{Capmul}^{MC}$:

- **Pi_FPl5:** Fifth component of the autocorrelation vector of pi Fukui(+) indices.
- **SolFactor:** Universal salt solubility factor based on S+Sw model.
- **N_CYPAtoms:** Number of potential atoms that can be oxidized by CYP P450 enzymes.
- **EEM_F4:** Fourth component of the autocorrelation vector of sigma Fukui indices.
- **Pi_FPl3:** Third component of the autocorrelation vector of pi Fukui(+) indices.

- **NPA_Q6:** Sixth component of the autocorrelation vector of estimated NPA partial atomic charges (6, 7).
- **MlogP:** Moriguchi estimation of log P (8).
- **MolVol:** Liquid molar volume (cm^3/mol) at the normal boiling point is based on Schroeder's method.
- **NPA_Q1**: First component of the autocorrelation vector of estimated NPA partial atomic charges.
- **S+S_Intrinsic**: Intrinsic water solubility (mg/mL) based on S+Sw and S+pH_Satd models.
- **EqualEta:** Equalized molecular hardness.
- **ΔH_{fus}**: - Enthalpy of fusion in kJ/mol obtained from literature.
- **M_CX**: Summation of numbers of carbon and halogen atoms weighted by C:1.0, F:0.5, Cl:1.0, Br:1.5, and I:2.0.
- **Pi_MinQ**: Minimum Hückel pi atomic charge.
- **N_Electr**. Total number of electrons in a molecule.

Variables in MLP-ANN Predicting aDS $sLBF_{Maisine}^{LC}$

- **N_Bonds**: Number of bonds.
- **Pi_FPl1:** First component of the autocorrelation vector of pi Fukui(+) indices.
- **T_Rads**: topological equivalent of Rads_3D.
- **MaxQ:** Maximal PEOE Partial Atomic Charge.
- **N_Atoms**: Number of atoms.
- **Pi_FMi1**: First component of the autocorrelation vector of pi Fukui(-) indices.
- **HBDch**: Sum of Estimated NPA Partial Atomic Charges on HB Donor Hydrogens.
- **F_AromB**: Aromatic bonds as fraction of total bonds.
- **NPA_Q2**: Second component of the autocorrelation vector of estimated NPA partial atomic charges.
- **SsssCH**: Atom-type E-state index for >CH- groups.
- **NPA_Q5**: Fifth component of the autocorrelation vector of estimated NPA partial atomic charges.

Figure S2. Predicted by Residual plots for $sLBF_{Capmul}^{MC}$ and $sLBF_{Maisine}^{LC}$ using PLS and ANN modelling.

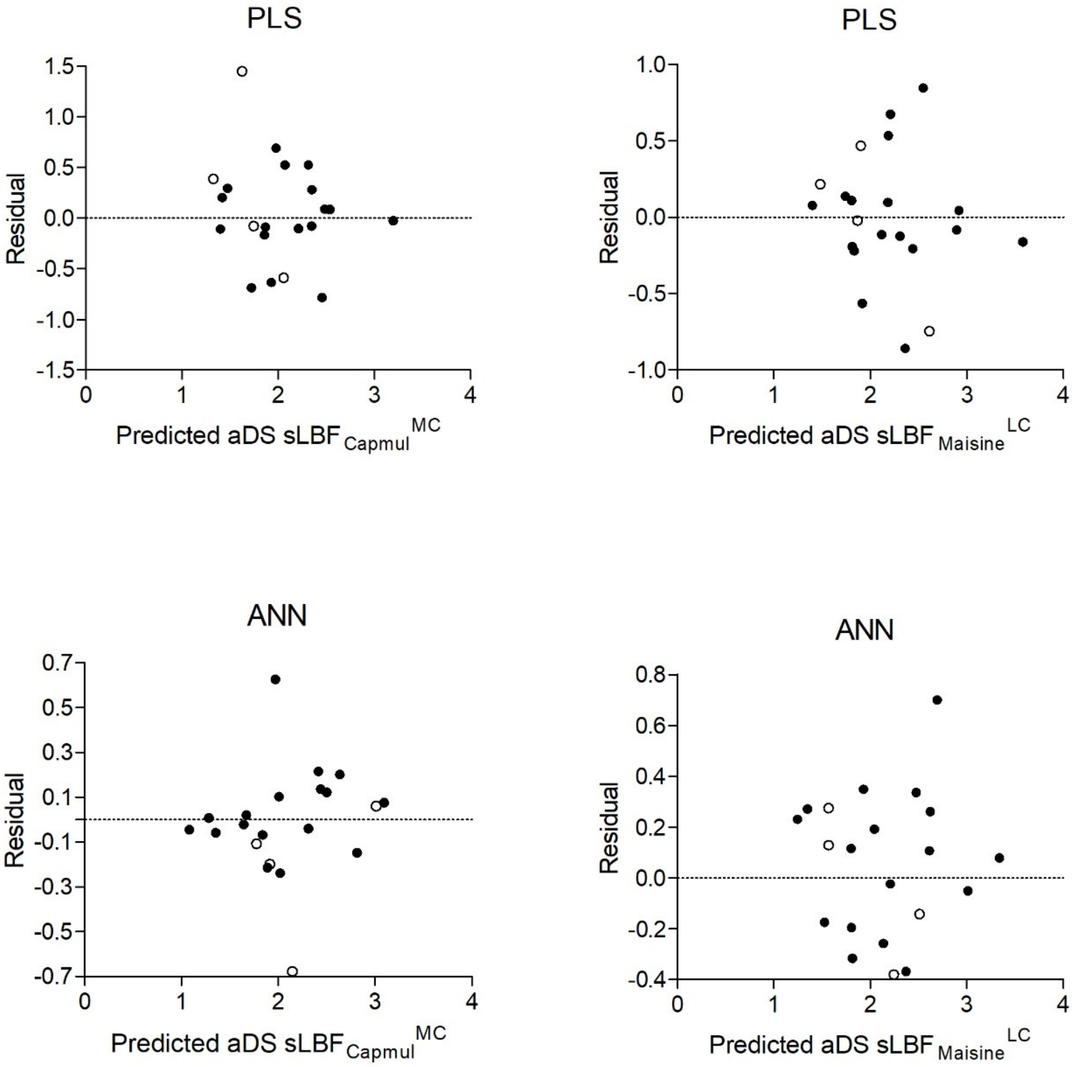

References:

1. Ilie, A.R.; Griffin, B.T.; Kolakovic, R.; Vertzoni, M.; Kuentz, M.; Holm, R. Supersaturated lipid-based drug delivery systems - exploring impact of lipid composition type and drug properties on supersaturability and physical stability. *Drug Dev Ind Pharm*. **2020**, *46*, 356-64, doi: 10.1080/03639045.2020.1721526.
2. Zhao, Y.H.; Abraham, M.H.; Zissimos, A.M. Determination of McGowan Volumes for Ions and Correlation with van der Waals Volumes. *Journal of Chemical Information and Computer Sciences*. **2003**, *43*, 1848-54, doi: 10.1021/ci0341114.
3. Kier, L.B.; Hall, L.H. An Electrotopological-State Index for Atoms in Molecules. *Pharmaceutical Research***.** **1990**, *7*,.801-7, doi: 10.1023/a:1015952613760.
4. Fukui, K.; Yonezawa, T.; Shingu, H.A. Molecular Orbital Theory of Reactivity in Aromatic Hydrocarbons. *The Journal of Chemical Physics*. **1952**, *20*, 772-25, doi: 10.1063/1.1700523.
5. Fradera, X.; Solà, M. Second-order atomic Fukui indices from the electron-pair density in the framework of the atoms in molecules theory. *J Comput Chem*. **2004**, *25*, 439-46, doi: 10.1002/jcc.10396.
6. Gasteiger, J.; Marsili, M. Iterative partial equalization of orbital electronegativity—a rapid access to atomic charges. *Tetrahedron*. **1980**, *36*, 3219-28, doi: 10.1016/0040-4020(80)80168-2.
7. Geidl, S.; Bouchal, T.; Raček, T.; Svobodová, Vařeková, R.; Hejret, V.; Křenek, A., et al. High-quality and universal empirical atomic charges for chemoinformatics applications. *Journal of Cheminformatics*. **2015**, *7*, 59, doi: 10.1186/s13321-015-0107-1.

8. Moriguchi, I.; Hirono, S.; Nakagome, I.; Hirano, H. Comparison of Reliability of log P Values for Drugs Calculated by Several Methods. *Chemical & Pharmaceutical Bulletin*. **1994**, *42*, 976-8, doi: 10.1248/cpb.42.976.
9. Todeschini, R.; Consonni, V. Molecular Descriptors for Chemoinformatics : Volume I & II Wiley-VCH Verlag GmbH & Co. *KGaA* **2009**.
